# The spectrum of opportunistic infections and malignancies among women on antiretroviral therapy in Ethiopia

**DOI:** 10.1080/22221751.2023.2271065

**Published:** 2023-10-12

**Authors:** Yimam Getaneh, Fentabil Getnet, Abdur Rashid, Li Kang, Qingfei Chu, Sisi Li, Feng Yi, Yiming Shao

**Affiliations:** a State Key Laboratory for Diagnosis and Treatment of Infectious Diseases, National Clinical Research Center for Infectious Diseases, Collaborative Innovation Center for Diagnosis and Treatment of Infectious Diseases, The First Affiliated Hospital, College of Medicine, Zhejiang University, Hangzhou, People’s Republic of China; b Ethiopian Public Health Institute, Addis Ababa, Ethiopia; c Takemi Program in International Health, Harvard T.H. Chan School of Public Health, Boston, MA, USA; d T.H. Chan School of Public Health, Boston, MA, USA; e School of Medicine, Nankai University, Tianjin, People’s Republic of China; f State Key Laboratory for Infectious Disease Prevention and Control, National Center for AIDS/STD Control and Prevention, Chinese Center for Disease Control and Prevention, Beijing, People’s Republic of China

**Keywords:** Incidence, mortality, opportunistic infections, malignancy, HIV/AIDS

## Abstract

*Background*: Women are disproportionally affected by HIV and are at greater risk of OIs and malignancies. This study investigated the spectrum of OIs and malignancies among women living with HIV (WLHIV) in Ethiopia. *Methods*: A retrospective longitudinal study was conducted among 3,817 WLHIV on Antiretroviral Treatment (ART) from 2007 to 2019 in Ethiopia. Data on OIs, malignancies, and clinical characteristics were retrieved from medical records. Prevalence, incidence, and mortality rates were measured over 13 years of follow-up. The Kaplan-Meier survival curve and multivariable Cox-proportional hazard regression were used to estimate incidence and identify predictors of OIs and malignancies, respectively. *Result*: The pooled prevalence of OIs or malignancies was 47%. OIs accounted for 39% and malignancies were 23.3%. The incidence of OIs and malignancies were 19.4 (95%CI:9.2-29.5) and 11.5 (95%CI:2.2-20.8) per 100 person-years of observation, respectively. Incidence of candidiasis was 14.1 per 100 person-years followed by Lymphoma (11.9), Cervical Cancer (11.5), and TB (10.9). The risk of OIs was higher in patients with baseline CD4 count ≤500 cells/mm3 [AHR (95%CI): 2.0 (1.8-2.3)] and VL>1000 copies/ml [AHR (95%CI): 1.5 (1.1-1.9)]. Overall, 24.6% died, and of them, 62.2% had a history of OIs or malignancies. The risk of death was higher among WLHIV with a history of Toxoplasmosis, TB and PCP. *Conclusion*: OIs and malignancies was recently increasing and two-thirds of all deaths were associated with these co-morbidities. The high rates of OIs and malignancies were attributed to poor treatment outcomes. The finding calls to enhance the diagnosis and treatment of these co-morbidities.

**Abbreviations:**

AIDS: acquired immune deficiency syndrome; CI: confidence interval; EPHI: Ethiopian Public Health Institute; HAART: highly active antiretroviral therapy; HIV: human immunodeficiency virus; HR: hazard ratio; Mg/dl: milligram per deciliter; TB: tuberculosis; PCP: pneumocystis carinii pneumonia; ZJU: Zhejiang University

## Background

Opportunistic infections (OIs) and malignancies are common in individuals with weakened immune systems, mainly among people living with HIV (PLWHIV) and malnourishment [1–3]. These conditions contribute to more than 90% of HIV/AIDS-related deaths [1]. Women were also reported to be disproportionally affected by OIs [2]. Moreover, malignancies are the most common comorbidities among women in low- and middle-income countries (LMICs), particularly cervical cancer and lymphoma [3–5]. Moreover, women are disproportionally affected by the HIV epidemic with a prevalence of 4.2% compared to males (1.2%) in urban Ethiopia [6]. Malnutrition among women was also reported to be significantly higher (33.2%) than in males (19.3%) [7].

Despite advances in HIV diagnosis and treatment, OIs, mainly Tuberculosis (TB), oral candidiasis, Herpes zoster, Toxoplasmosis, and *Pneumocystis Pneumonia* (PCP), remain major causes of morbidity and mortality in HIV/AIDS patients in LMICs [8]. Recent studies in Ethiopia have shown an increasing trend of OIs among PLHIV [9,10]. On the other hand, malignancies have also been among the leading causes of mortality in PLHIV which accounted for 25%–40%. The commonest malignancies among WLHIV are Lymphoma and cervical cancer [11]. Cervical cancer is the second leading cause of cancer-related illness and deaths among women with an estimated 570,000 cases and 311,000 deaths per year [5], and about 90% of cases and deaths occur in LMICs [12]. Ethiopia is among the top ten countries globally and second in Eastern Africa with over six thousand cases and four thousand deaths annually [9]. HIV is associated with Human Papilloma Virus (HPV) infection augments the progression, transformation, and aggressiveness of premalignant lesions due to HPV infection [9].

Hence, early detection of OIs, cervical cancer, and lymphoma among PLHIV is essential to prevent complications, improve treatment outcomes, and ultimately prolong survival. In recent years, efforts have been made to integrate the diagnosis and treatment of OIs and malignancies in HIV care and treatment in Ethiopia. Moreover, the country has been working on screening cervical cancer using Visual Inspection using Acetic Acid (VIA) since 2016 and has been moving towards universal HPV vaccination for young adults (15–24 years old) since 2020 [10]. However, there is no clear programme strategy for screening other non-communicable diseases among PLHIV in Ethiopia. The country also had a guideline on integrating TB-HIV services during the clinical management of PLHIV. This includes diagnosis of active TB for PLHIV and providing INH/IPT and CPT prophylactic.

Despite these efforts, there is limited evidence on the magnitude of OIs and malignancies among WLHIV in Ethiopia. Hence, this study aimed to describe the spectrum (i.e. incidence and prevalence) of OIs and malignancies and associated mortality among WLHIV in Ethiopia.

## Methods

### Study design and setting

This study was part of a nationwide cohort study on HIV-1 treatment and drug resistance at 63 health facilities in Ethiopia between 2007 and 2019 [8]. Of the 63 facilities, 20 referral hospitals and university hospitals that had OIs and malignancy diagnosis services were included in this analysis.

### Population, sample size, and sampling

**Figure 1.**
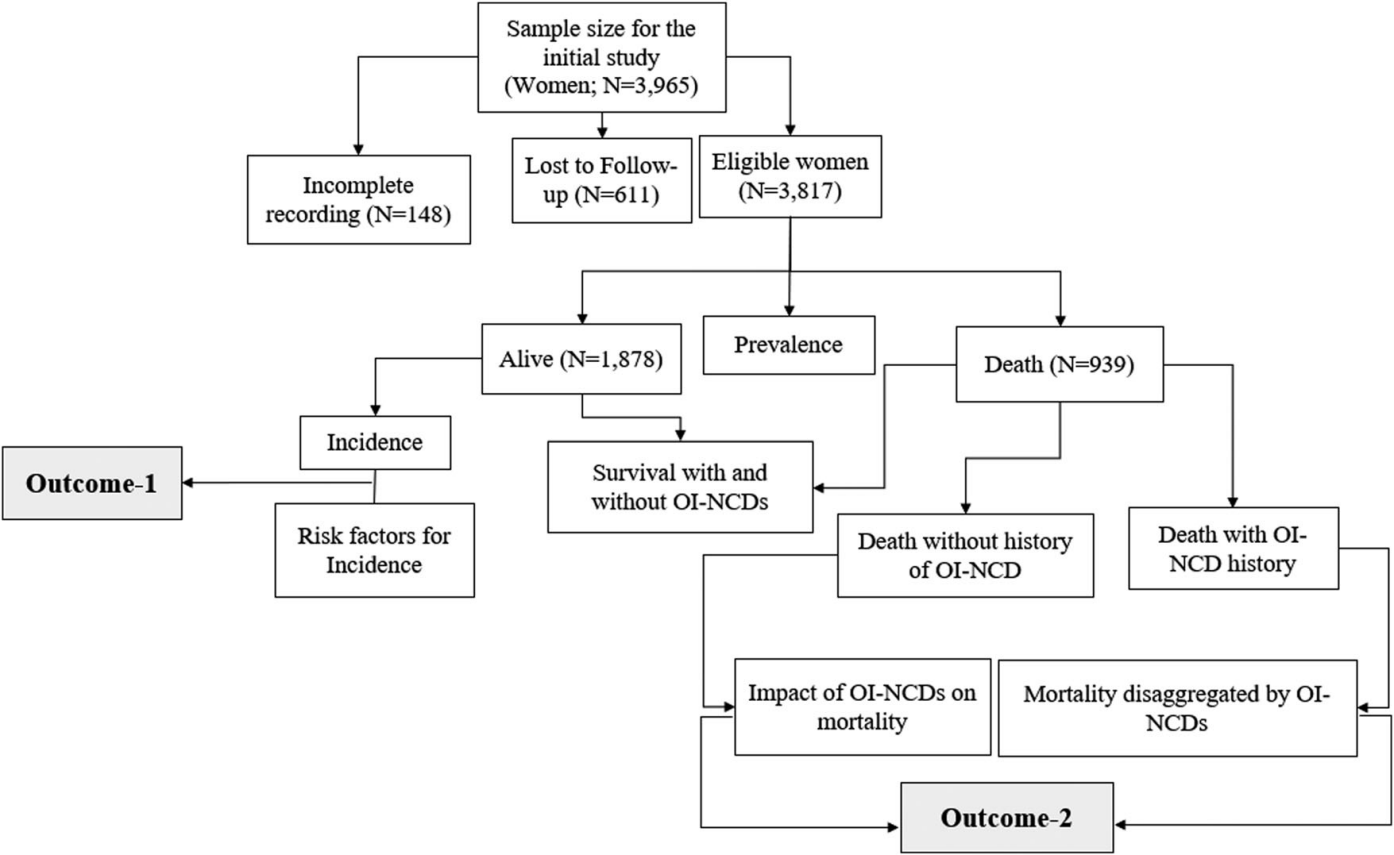
Sampling, sample size, and eligibility criteria among WLHIV in Ethiopia (2007–2019).

We included all Adult WLHIV (≥15 years old) who were enrolled between January and December 2007 and on follow-up for any length of time until December 2019 at the 20 hospitals with diagnosis services for OIs and malignancy. We excluded WLHIV who were diagnosed with any OIs and malignancies at baseline screening. We also excluded patients with incomplete records of baseline information such as CD4 count, WHO clinical stage, date of Highly Active Antiretroviral Therapy (HAART) initiation, functional status, and status of OIs or malignancies. Loss to follow-up was also excluded from the analysis as of the date of their classification as lost. Moreover, accordingly, a total of 3,817 WLHIV were found eligible and included in the analysis (Figure 1).

### Diagnosis of OIs and malignancies

At recruitment for the study, we evaluated and excluded those who were not eligible for this study and included as much as possible baseline information. We also have evidence of time to diagnosis. However, our analysis started from the time of HAART initiation since the test and treatment were not implemented by that time and the eligibility criterion for HAART initiation was a CD4 count of <500 cells/mm^3^. This may imply the high burden of OIs and malignancies at an early stage of HAART initiation.

TB was diagnosed using AFB microscopic or GeneXpert, whereas other common OIs (i.e. oral candidiasis, Toxoplasmosis, PCP, and Herpes zoster) were diagnosed or defined according to the national guideline for syndromic/clinical diagnosis of OIs during HIV care [13]. **HIV-related malignancies**: common malignancies among WLHIV (Cervical cancer and Lymphoma) were targeted in this study. Cervical cancer was determined using Visual Inspection with Acetic Acid (VIA) or Papanicolaou (PAP) smear complemented with clinical evidence, as recorded by the attending physician. Lymphoma was also defined per the clinical definitions according to the national comprehensive ART guideline (https://www.childrenandaids.org/sites/default/files/201805/Ethiopia_Nat%20Guidelines%20ART_2017.pdf); https://www.afro.who.int/sites/default/files/201904/National%20Comprehensive%20HIV%20Care%20%20Guideline%202018.pdf [13].

## Measurement

**Incidence**: Time from ART initiation to the occurrence of any OIs or malignancies among WLHIV from recruitment until death or lost-follow-up or end-of-follow-up.

**Prevalence**: the occurrence of any OIs or malignancies among WLHIV at any time during the follow-up period.

**Mortality**: death of women with a history of OIs or HIV-associated malignancies divided by overall death.

**Lost to follow-up**: Patients missing their follow-up visits according to their appointment schedule for more than 3 months as recorded on the appointment schedule.

## Data collection

Data were extracted from medical and electronic records using an abstraction tool designed for this purpose and adapted from the national HIV care and ART follow-up intake records from March to May 2020. We used the Open Data Kit (ODK) programmed in tablet computers and integrated into the Ethiopian Public Health Institute (EPHI) server for real-time data capture. Data were extracted on occurrences of OIs, malignancies, deaths, and explanatory variables including socio-demographic (i.e. age, sex, residence, marital status, and occupation); Clinical and laboratory (i.e. clinical stage, CD4 count, and functional status), and medication-related (i.e. HAART regimen and adherence). Data completeness and consistency were double-hecked on 15% of randomly included records. The predictor variables had been recorded at baseline and occurrences of OIs, malignancies, and death were registered during follow-up period.

## Data analysis

Descriptive statistics were employed to summarize the incidence and percentage of OIs and malignancies, and frequencies and median/mean to summarize key socio-demographic and clinical characteristics. The Kaplan-Meier curve was used to demonstrate the incidence of OIs and malignancies per 100 person-year observations. A cox-proportional hazard regression model was fitted to identify the predictors of OIs and malignancies among WLHIV. Variables with *p*-values ≤0.20 in bivariate analysis were included in the final multivariable model. In addition, a generalized Log rank test was used to compare the OI or malignancy survival time between different categorical predictor variables. Adjusted Hazard Ratio (AHR) with a 95% confidence interval was used to determine effect sizes at *p* < 0.05. STATA Version 17 was used for analysis.

## Result

### Socio-demographic and clinical characteristics

A total of 3817 WLHIV were included in the analysis. The mean age of participants at baseline was 38 (±10) years old. Majorities were urban by residence (93.2%), ever married (89.5%), and aged 26–45 years (74.5%). At baseline, 63.8% had a CD4 count of >500 cells/mm^3^ and were on WHO clinical stage I (91%) (Supplementary Table 1).

### Prevalence and incidence of OIs and HIV-associated malignancies

The combined prevalence of OIs or malignancies was 47% (95% CI: 40.8–53.2) among WLHIV over the 13 years of follow-up in Ethiopia. The prevalence of OIs and malignancies was 39% and 23.3%, respectively. Oral candidiasis had the highest prevalence (14.2%) among OIs followed by TB (11.3%), Herpes zoster (9.6%), PCP (2.3%), and Toxoplasmosis (1.6%). The prevalence of Lymphoma and cervical cancer was also 13.3% and 11.9%, respectively (Table 1).

**Table 1.** Prevalence of OIs and HIV-associated malignancies disaggregated by clinical characteristics among WLHIV in Ethiopia (2007–2019).

Characteristics	Oral candidiasis (%)	Herpes (%)	PCP (%)	Toxoplasmosis (%)	TB (%)	Overall OIs (%)	Cervical cancer (%)	Lymphoma (%)	Overall malignancies (%)
**Residency**									
Urban	14.3	9.8	2.2	1.4	11.0	38.8	11.9	13.2	25.1
Rural	13.5	7.7	2.7	3.8	14.2	41.9	11.5	14.2	25.7
**Age**									
≤24	16.5	8.7	2.6	1.7	12.1	41.6	13.0	11.3	24.3
25–34	13.1	9.9	2.5	1.6	11.3	38.3	7.7	12.6	20.3
35–44	14.9	9.9	2.1	1.6	10.9	39.4	14.9	12.1	27.0
45–54	16.3	8.6	1.7	1.7	12.1	40.4	12.6	16.3	28.9
55–64	8.4	9.6	3.0	1.2	11.4	33.7	19.3	13.9	33.2
65–74	15.2	10.9	0.0	2.2	6.5	34.8	13.0	13.4	26.4
≥75	33.3	0.0	0.0	0.0	16.7	50.0	16.7	80.0	96.7
**Adherence**									
Poor	7.1	14.3	0.0	0.0	7.1	28.6	10.7	11.7	22.4
Fair	5.0	15.0	0.0	0.0	15.0	35.0	9.1	18.2	27.3
Good	13.0	10.4	2.3	1.3	8.6	35.7	16.7	16.7	33.4
Very good	14.5	9.5	2.3	1.7	11.6	39.5	10.7	16.0	26.7
**Functional status**									
Ambulatory	14.3	9.7	2.3	1.6	11.2	39.0	14.1	11.1	25.2
Bedridden	8.9	6.7	0.0	4.4	17.8	37.8	11.5	13.2	24.7
**Clinical stage**									
I	14.2	9.6	2.2	1.4	11.2	38.7	14.0	14.0	28.0
II	13.9	9.8	2.8	3.2	12.3	42.0	6.6	8.2	14.8
III	18.2	3.0	3.0	3.0	3.0	30.3	14.2	12.3	26.5
IV	16.7	50.0	0.0	16.7	16.7	100.0	11.1	13.0	24.1
**Viral load**									
≤1000	14.2	9.5	2.2	1.5	10.5	37.9	12.6	15.1	27.7
>1000	14.4	11.3	3.4	2.4	20.3	51.9	19.3	14.5	33.8
**CD4 count**									
≤500	16.3	14.2	2.9	3.0	15.7	52.1	13.0	21.7	34.7
>500	13.1	7.1	1.9	0.8	8.7	31.5	16.7	0.0	16.7
**Overall (%)**	14.2	9.6	2.3	1.6	11.3	39.0	11.9	13.3	25.2

The incidence of OIs and malignancies among WLHIV was 19 (95% CI: 9–29) and 11 (95% CI: 2–20) per 100 person-years of observation, respectively. Of all morbidities, oral candidiasis was the highest (14.1 per 100 person-years) followed by Lymphoma (11.9), Cervical Cancer (11.5), and TB (10.9) (Table 2).

**Table 2.** Prevalence of OIs or HIV-associated malignancies among WLHIV in Ethiopia (2007–2019).

Year	Oral candidiasis		Herpes zoster		PCP		Toxoplasmosis		TB		Cervical cancer		Lymphoma	
	%	Incidence	%	Incidence	%	Incidence	%	Incidence	%	Incidence	%	Incidence	%	Incidence
2007	28	0.02	21.7	0.015	9.5	0.007	3.9	0.005	20.1	0.012	13.5	0.01	27.6	0.018
2008	22.8	0.035	16.3	0.026	0.7	0.007	3.8	0.007	18.3	0.025	9	0.017	21.8	0.034
2009	16.7	0.046	11.5	0.034	2.4	0.009	2.4	0.009	17.4	0.036	10.5	0.025	12.5	0.042
2010	14.7	0.055	9.8	0.04	3	0.011	1.5	0.009	15.4	0.044	9.8	0.032	9.8	0.047
2011	13.3	0.065	8.1	0.047	3.2	0.013	1.1	0.01	14.7	0.056	11.2	0.04	14.4	0.058
2012	8.9	0.077	7.9	0.057	0.6	0.014	0.4	0.01	3.5	0.059	11.2	0.055	6.8	0.066
2013	14.8	0.1	7.7	0.07	1.8	0.016	1.8	0.013	11	0.074	11.5	0.075	8.1	0.079
2014	12.3	0.11	2.7	0.073	1.4	0.017	1.9	0.014	3	0.076	14.5	0.091	5.2	0.085
2015	2.7	0.111	5	0.076	2.7	0.018	2.3	0.015	1.8	0.078	13.6	0.099	10	0.09
2016	7.1	0.113	3.9	0.076	2.4	0.018	1.6	0.015	10.2	0.08	14.1	0.103	8.6	0.091
2017	27	0.123	26.2	0.086	5.7	0.021	2.5	0.016	30.3	0.089	14.8	0.108	23	0.099
2018	34.7	0.138	12.8	0.093	5.1	0.023	4.6	0.017	27	0.102	11.7	0.113	25.5	0.113
2019	12.2	0.141	24.4	0.1	3.3	0.024	2.2	0.018	38.9	0.109	11.1	0.115	33.3	0.119
Overall	16.5	0.087	12.1	0.061	3.2	0.01	2.30	0.012	16.2	0.065	12.0	0.068	15.8	0.072

### Trends of OIs and HIV-associated malignancies

**Figure 2.**
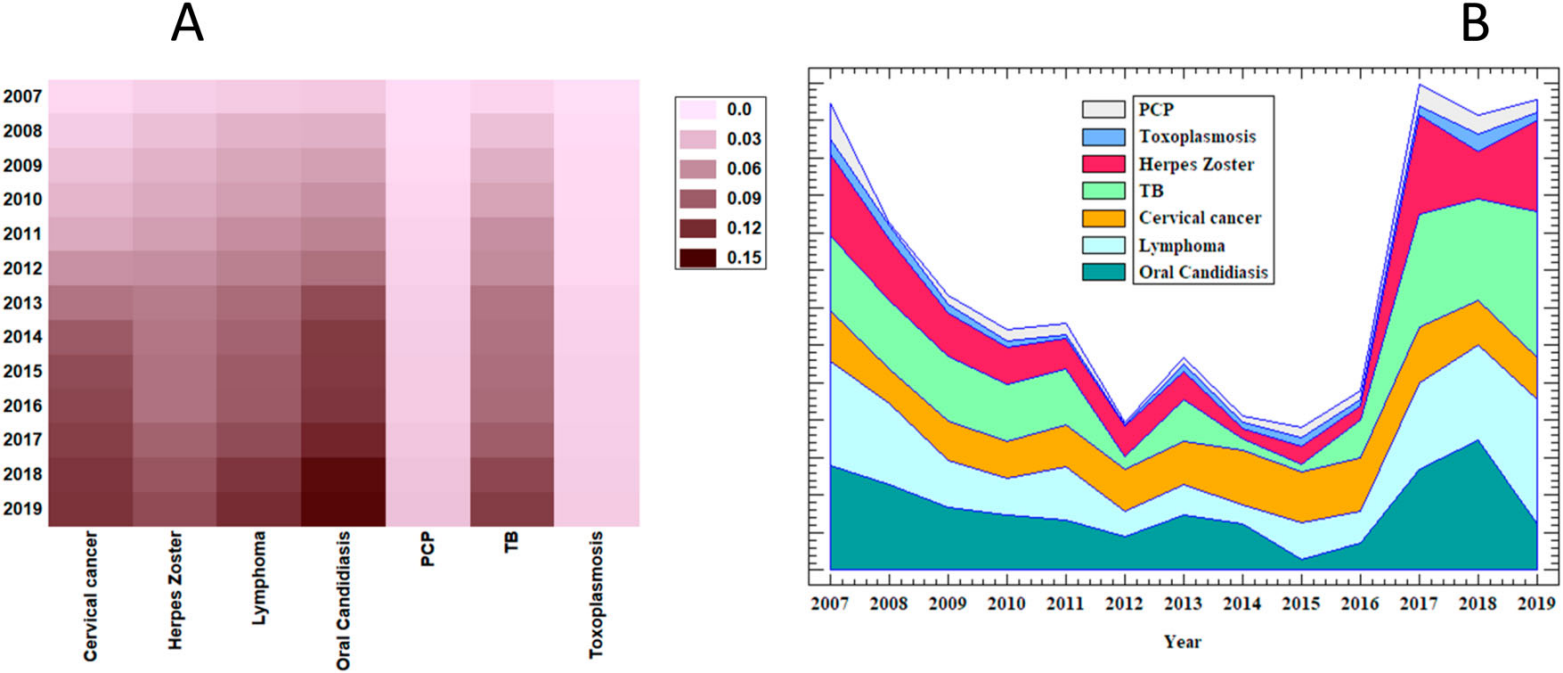
Trends of Incidence and prevalence of Opportunistic infection and HIV-associated malignancies among WLHIV in Ethiopia (2007–2019): (A) Incidence of OIs and HIV-associated malignancies (per 100-person year observation) (B) Trends of prevalence OIs and HIV-associated malignancies.

The overall prevalence of OIs during the first year of follow-up was 62.8% which declined overtime until the nineth year of follow-up (19.1%) and then significantly increased (*P* < 0.001) to 39% by the last year of follow-up. The prevalence of oral candidiasis declined from 28.0% at baseline to 12.2% by 2019, PCP from 9.5% to 3.3%, and Toxoplasmosis from 2.9% to 2.2%. Herpes Zoster was relatively constant (i.e. 21.7% by 2007 vs. 24.4% by 2019). TB increased from 20.1% in 2007 to 38.9% in 2019, and lymphoma increased from 27.6% to 33.3% (Table 2 and Figure 2).

### Predictors of OIs and HIV-related malignancies

After adjusting for confounders, patients with CD4 count ≤500 cells/mm^3^ at baseline had higher hazards of OIs [AHR (95% CI) = 2.3 (2.0–2.6)] and malignancies [AHR (95% CI) = 1.2 (1.04–1.4)]. Patients with VL > 1000 copies/ml had also a higher risk of OIs [AHR (95% CI) = 1.4 (1.1–1.7)] and malignancies [AHR (95% CI) = 1.8 (1.4–2.4)] (Table 3).

**Table 3.** Predictors of OIs and HIV-related malignancies among WLHIV in Ethiopia (2007–2019).

Variable		Malignancies only (AHR)				OIs (AHR)				All malignancies or OIs (AHR)			
		*P*-value	AHR	95% CI		*P*-value	AHR	95% CI		*P*-value	AHR	95% CI	
CD4 count (cells/mm^3^)	≤500	0.01	1.21	1.04	1.42	0.00	2.28	1.99	2.62	0.00	2.04	1.78	2.34
	>500	Ref.											
Haemoglobin (g/dl)	≤12	0.06	0.69	0.47	1.01	0.06	0.69	0.47	1.01	0.09	0.73	0.51	1.05
	>12	Ref.											
Viral load (copies/ml)	≤1000	Ref.											
	>1000	0.00	1.82	1.40	2.36	0.01	1.35	1.05	1.73	0.00	1.47	1.14	1.89
Functional status	Alive	0.00	2.87	1.31	4.38	0.01	2.11	1.18	3.21	0.00	3.12	1.83	4.32
	Death	Ref.											

### Mortality associated with OIs and malignancies

**Figure 3.**
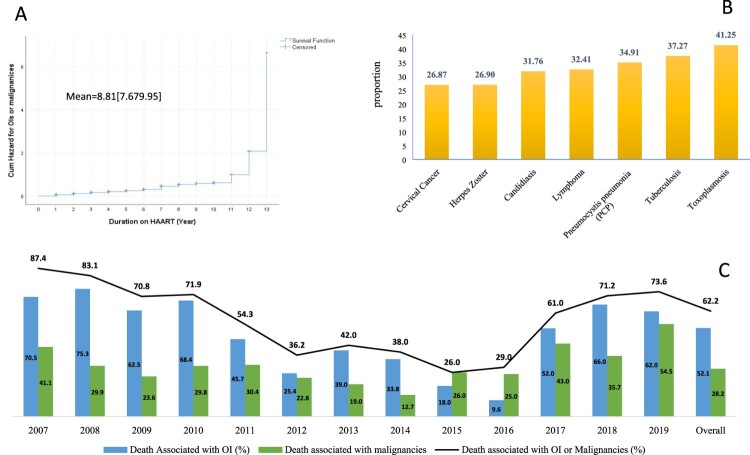
Mortality associated with OIs and malignancies among WLHIV in Ethiopia (2007–2019). (A) Incidence of OIs and HIV-associated malignancies (B) Proportion of mortality disaggregated by OIs and malignancies (C) Trends of Mortality associated with OIs and malignancies.

**Figure 4.**
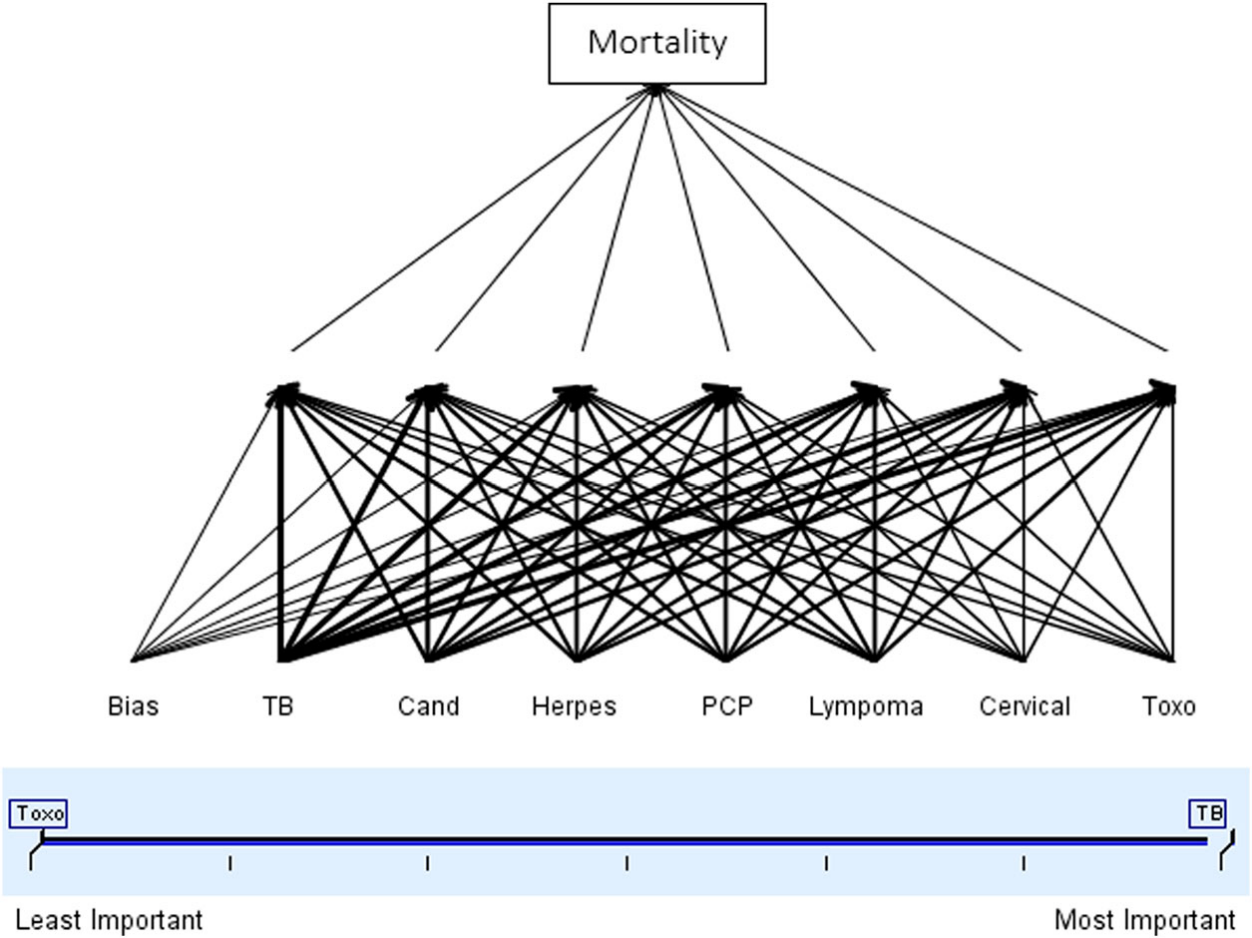
OIs or malignancies associated with mortality among WLHIV in Ethiopia by their importance.

Overall, 24.6% of WLHIV died during the 13 years of follow-up of whom 62.2% had a history of at least one OI or malignancy. Mortality was higher among WLHIV with a history of multiple infections (35.8%), *p* < 0.05. Toxoplasmosis was the most prevalent infection among dead cases (41.2%) followed by TB (37.3%) and PCP (34.9%). Among OIs or malignancies associated with death, 32.5% had a history of oral candidiasis followed by TB (32%) and Lymphoma (28%) (Figure 3 and Table 4). The risk of death was significantly higher among WLHIV diagnosed with Toxoplasmosis [AHR (95% CI) = 2.1 (1.3–3.41)], TB [AHR (95% CI) = 2.1 (1.7–2.6)], and PCP [AHR (95% CI) = 1.4 (1.2–2.3)] (Table 4). As indicated in Figure 4, the most important predictor of mortality was TB while the least was Toxoplasmosis which might be associated with its low prevalence despite the high mortality among Toxoplasmosis-infected patients.

**Table 4.** The risk of OIs and HIV-associated malignancies on mortality among WLHIV in Ethiopia (2007–2019).

OI or HIV-related malignancy	*P*-value	AHR	95% CI	
			Lower	Upper
Oral candidiasis				
Yes	0.00	1.21	1.04	1.98
No	Ref.			
Herpes zoster				
Yes	0.02	1.32	1.04	1.69
No	Ref.			
Pneumocystis pneumonia (PCP)				
Yes	0.01	1.39	1.21	2.32
No	Ref.			
Toxoplasmosis				
Yes	0.00	2.14	1.34	3.41
No	Ref.			
Tuberculosis				
Yes	0.00	2.11	1.72	2.59
No	Ref.			
Cervical cancer				
Yes	0.30	1.12	0.89	1.41
No	Ref.			
Lymphoma				
Yes	0.00	1.54	1.26	1.90
No	Ref.			

## Discussion

This study showed that the pooled prevalence of OIs or malignancies among WLHIV during the 13 years of follow-up was 47% (39% for OIs and 23.3% for malignancies). The most prevalent OIs were oral candidiasis (14.2%), TB (11.3%) and Herpes zoster (9.6%), whereas the prevalence of cervical cancer and Lymphoma was 11.9% and 13.3%, respectively. The Incidence of OIs or malignancies was 19.7 per 100 person-years observation, respectively. Oral candidiasis was the most incident case with 14 per 100 person-years followed by lymphoma (12), Cervical Cancer, and TB (11). Patients with CD4 count <500 cells/mm^3^ and VL > 1000 copies/ml had a higher risk of acquiring OIs and malignancies. A quarter of patients died during follow-up and significantly associated with Toxoplasmosis, TB, and PCP.

The overall prevalence of OIs in the current study was almost double the findings from Northwest Ethiopia [14] but much lower than another report from Northeast Ethiopia [15]. Moreover, the increasing trends of oral candidiasis and Tuberculosis after ART initiation were in line with the report in Northeast Ethiopia [15]. On the other hand, the incidence of OIs in this study was higher than previous reports in Northeast [15] and northcentral Ethiopia [16], China [17], and South Korea [18], comparable with a report in Senegal [8], and lower compared to the finding in India [19]. This variation might be attributable to variations in length of follow-up, sample sizes, study population, and socio-economic and healthcare settings, for example, the previous studies on Ethiopia were conducted on single health facilities. Specifically, the prevalence of oral candidiasis (14.2%) and Herpes zoster (9.6%) in this study was relatively lower compared to those who were HAART naïve in LMIC which was reported to be 19.1% [95% CI, 13.0%–27.3%] and 14.4% [95% CI, 6.7%–13.2%] [8], respectively. This could be explained by the importance of HAART in reducing OIs. Moreover, 11.3% prevalence of TB in this study was significantly higher compared to prevalence in the general population in Ethiopia which was reported to be 0.19% [95% CI: 0.12%–0.28%] [20,21]. This highlights a 6-fold risk of TB among PLHIV compared to the general population in the country.

The overall prevalence of malignancies was relatively higher compared to the findings [4,8,11,18,20,21]. A relatively higher prevalence of cervical cancer in this study could be explained by the limited availability of HPV vaccination, cervical cancer screening programmes, and a high prevalence of human papillomavirus (HPV) infection among HIV-infected women in Ethiopia [6,10]. A systematic review estimated a higher prevalence of cervical cancer and reported the risk of cervical cancer increased in WLHIV (RR = 6.07, 95% CI = 4.40,8.37). Globally, 5.8% (95% CI = 4.6,7.3) of new cervical cancer cases in 2018 (33,000 new cases, 95% CI = 26,0,42,0) were diagnosed in and 4.9% (95% CI = 3.6,6.4) were attributable to HIV infection (28,000 new cases, 20,000–36,000). Moreover, the PEPFAR Ethiopia reported age-standardized incidence rate of HPV among the general population in Ethiopia was 21.5 per 100,000 person-years equivalent to 9.6% prevalence by 2020 [22]. Our report is significantly higher compared to these estimates which could be due to the fact that our study population had a 13 years of follow-up. The previous report also highlighted most affected regions by cervical cancer were southern Africa and Eastern Africa. In southern Africa, 63.8% of WLHIV had cervical cancer, compared to 27.4% in Eastern Africa [9]. This was also relatively higher compared to our report which could be explained by the scope of the study which covered East African countries and may also be due to the fact that cervical cancer screening is yet a challenge and under-estimated by the different studies. Our finding, therefore, calls for strengthening cervical cancer screening among WLHIV, particularly among those who had more than 10 years of HAART experience women who had the highest incidence rate. The overall prevalence of Lymphoma among WLHIV in Ethiopia was 13.26%. Moreover, Lymphoma among the general population in Ethiopia accounted for 4.9% [23]. This report was also higher than a systematic review conducted in India (6.5%) [24]. In this study, Lymphoma among elderly WLHIV was high which could be explained by the immune deterioration of the elderly that could exacerbate the development of lymphoma and other malignancies. This finding highlights the importance of lymphoma diagnosis among the geriatric population.

Our analysis shows that WLHIV with CD4 count <500/mm^3^ had a two-fold higher risk of developing OIs which is consistent with previous studies [8–10,17,18,25,26]. Similarly, a higher risk of OIs among those with VL > 1000 copies/ml [17,25,27]. The result of the association between CD4 count and incident OIs confirms the known biological relationship between low CD4 count and OIs [28]. The higher risks of malignancies among patients with CD4 count <500/mm^3^ and VL > 1000 copies/ml were also supported by many studies [4,11,18,24,29]. Hence, the impact of OIs and malignancies on treatment outcomes is inevitable. The re-increase of OIs and malignancies as of 2014 could be explained by a war in the country which limited access to health services and follow-up of PLHIV to the health facilities. This limited not only the HIV care and treatment but also factors in late diagnosis. The other reason may be, the limited attention on these co-morbidities in recent years.

The rate of mortality among WLHIV was consistent with previous studies in Ethiopia [30,31], and significantly higher rates of death in patients with multiple infections were also supported by various studies [11,25,32,33]. However, this study reported a two-third mortality associated with OIs or malignancies which was significantly high. This highlights the significant impact of these comorbidities among WLHIV in Ethiopia. The higher risks of death among WLHIV diagnosed with Toxoplasmosis and TB were consistent with previous studies [17,34–37].

Due to the limited information in the recordings, the different stages of cervical cancer and the types of lymphoma were not characterized. Nearly 20% of records were excluded due to lost-to-follow-up or incomplete records. This would affect the generalizability of findings since the missed cases would have special features that would affect the occurrence of OIs and malignancies. Moreover, this study was conducted at referral hospitals and university hospitals where most patients are urban and relatively high economic compared to rural WLHIV. The facilities had limited capacity during the earlier times to diagnose OIs and HIV-related malignancies and hence misclassification or misdiagnosis might happen. Since this study was concluded by the end of 2019, the burden of OIs and malignancies during after the era of COVID-19 was not part of this study.

## Conclusion

Nearly half of WLHIV develop either OIs or malignancies while the incidence rate was also increasing, particularly for TB and Toxoplasmosis. A quarter of WLHIV died during the 13 years of follow-up, of whom two-thirdss were associated with OIs or malignancies. The high rates of mortality associated with opportunistic infections were attributed to immunologic and virologic failures. The finding calls to enhance the diagnosis and treatment of OIs and malignancies particularly TB, Toxoplasmosis, and cervical cancer during the clinical management of WLHIV.

## Operational definition

**OIs**: common opportunistic infections (i.e. TB, Candidiasis, Toxoplasmosis, PCP, and Herpes zoster). TB was diagnosed using AFB microscopic or GeneXpert. Moreover, oral candidiasis, Toxoplasmosis, PCP, and Herpes zoster were defined per the clinical definitions according to the national comprehensive ART guideline on Ethiopia [13].

**Malignancies**: common malignancies among WLHIV (Cervical cancer and Lymphoma). Cervical cancer was determined using Visual Inspection with Acetic Acid (VIA) or PAP smear complemented with clinical evidence as recorded by the attending physician. Lymphoma was also defined per the clinical definitions according to the national comprehensive ART guideline on Ethiopia [13].

**Lost to follow-up**: Patients missing their follow-up visits according to their appointment schedule for more than 3 months as recorded on the appointment schedule.

WHO clinical stages, CD4 count, and VL suppression were described as per the national compressive ART guideline [13].

## Declarations

### Ethical considerations

Ethical approval was obtained from the Ethiopian Public Health Institute Scientific and Ethical Review Office (SERO) with approval number; EPHI-IRB-19110-2019. Confidentiality was respected during the abstraction of data by the use of a specific identification code for each enrolled patient number. Given the nature of the data collection was a medical record review, informed consent from the study participants was not applicable.

## Supplementary Material

Supplemental Material

## Data Availability

Since data analysis for other objectives is ongoing, the raw data can be obtained from the first corresponding author.
