# Supplementary material for: The spectrum of opportunistic infections and malignancies among women on antiretroviral therapy in Ethiopia
Source: Emerg Microbes Infect. 2023 Oct 12;12(2):2271065. doi: 10.1080/22221751.2023.2271065 (PMC10614708; doi:10.1080/22221751.2023.2271065)
Supplement: Supplemental Material [file TEMI_A_2271065_SM4759.docx]

**Suplimentary-Table-1**: Demographic and clinical characteristics of WLHIV in Ethiopia (2007-2019)

| Characteristics | | Total patients (N) | Percent (%) | OI or Malignancies (%) |
| --- | --- | --- | --- | --- |
| Residency | Urban | 3557 | 93.2 | 46.8 |
|  | Rural | 260 | 6.8 | 49.6 |
| Age (Binned) | ≤24 | 231 | 6.1 | 47.6 |
|  | 25-34 | 1474 | 38.6 | 45.4 |
|  | 35-44 | 1372 | 35.9 | 47.9 |
|  | 45-54 | 522 | 13.7 | 49.4 |
|  | 55-64 | 166 | 4.3 | 45.7 |
|  | 65-74 | 46 | 1.2 | 47.8 |
|  | 75-84 | 6 | 0.2 | 50.0 |
| Adherence | Poor | 28 | 0.7 | 57.1 |
|  | Fair | 40 | 1 | 52.5 |
|  | Good | 384 | 10.1 | 45.5 |
|  | Very good | 3365 | 88.2 | 47.1 |
| Functional status | Ambulatory | 3772 | 98.8 | 47.0 |
|  | Bedridden | 45 | 1.2 | 48.8 |
| Clinical stage | I | 3461 | 91 | 46.8 |
|  | II | 317 | 8.3 | 48.9 |
|  | III | 33 | 0.9 | 42.4 |
|  | IV | 6 | 0.2 | 100.0 |
| VL | ≤1000 | 3526 | 92.4 | 45.9 |
|  | >1000 | 291 | 7.6 | 61.7 |
| CD4 | ≤500 | 1382 | 36.2 | 58.9 |
|  | >500 | 2435 | 63.8 | 40.3 |
